# Variable bone mineral density reductions post-unicompartmental knee arthroplasty

**DOI:** 10.1007/s00167-014-3014-5

**Published:** 2014-04-27

**Authors:** Mahmut Tuncer, Rajesh Patel, Justin P. Cobb, Ulrich N. Hansen, Andrew A. Amis

**Affiliations:** 1 Department of Mechanical Engineering, Imperial College London, Exhibition Road, London, SW7 2AZ UK; 3 Department of Musculoskeletal Surgery, Charing Cross Hospital, Imperial College London, London, W6 8RF UK

**Keywords:** Unicompartmental knee arthroplasty, UKA, Bone density changes, BMD, DXA

## Abstract

**Purpose:**

Radiolucencies are commonly observed in unicompartmental knee arthroplasty (UKA) patients within 1 year of arthroplasty. The objective of the study was to identify how the bone mineral density (BMD) changes up to 1 year post-arthroplasty.

**Methods:**

Dual X-ray absorptiometry scans were obtained from 11 UKA patients at 10 days and 3, 6, and 12 months post-surgery. Patients were scanned in both anteroposterior and lateral knee orientations.

**Results:**

Most subjects saw a large decline in BMD in the first 6 months following surgery, followed by some recovery in bone mass. The biggest change occurred under the tibial intercondylar eminence, which decreased significantly by an average of 18 % at 6 months and was 15 % at 1 year. The average bone loss under the tibial tray was low; however, the bone loss at the anterior portion was higher with a significant average decrease of 14 %. There was no change in BMD under the tibial keel. There was significant bone loss of 13 % under the femoral component; the regions anterior and posterior to the central femoral implant peg both had significant bone loss of 14 %. The bone response between patients was very variable, with some patients losing bone steadily, and others gaining it rapidly after an early fall.

**Conclusions:**

While the overall reduction in BMD under both components was low, it was significant and there was substantial individual variation superimposed on this. Improving our understanding of this response to surgery may impact on prosthesis survival.

**Level of evidence:**

Therapeutic study: case series with no comparison group, Level IV.

## Introduction

There is increasing evidence that unicompartmental knee arthroplasty (UKA) can have reliable long-term performance [7, 14, 17, 18]. Early loosening is the most common reason for revision surgery [1, 2, 12, 17, 23], and stress shielding followed by bone resorption may contribute to the process. Radiolucencies are very commonly seen beneath mobile-bearing UKA, starting to occur within one year post-arthroplasty [19]. While most of these radiolucencies are claimed to be ‘physiological’, those that are thick with undefined borders have been linked to loosened implants [5]. There is a need to understand the bone density changes that occur beneath UKA components post-arthroplasty, to aid further development of their fixation.

Dual X-ray absorptiometry (DXA) scanning is commonly used to measure bone mineral density (BMD) and changes in BMD over time. Although numerous studies have been conducted on total knee arthroplasty (TKA) patients [3, 10, 13, 16, 25], only one DXA study has been conducted on UKA patients [24], examining fixed-bearing UKA up to 7 years post-surgery; they did not find significant changes in BMD beyond 1 year post-UKA. A further study [20] used CT slices to find almost no changes in overall tibial BMD post-UKA. There remains a need for more detailed information on bone changes in UKA, particularly for mobile-bearing prostheses.

Noting that most bone remodelling occurs within 1 year [4, 22, 24], it was hypothesised that there would be a loss of BMD within 1 year post-UKA; knowledge of such changes would aid work to improve the fixation design.

## Materials and methods

Following approval by the Charing Cross Hospital Research Ethics Committee (Ref 09/H0711/51), thirteen UKA patients were recruited over the course of 1 year. All surgeries were performed by a single consultant surgeon and his registrar.

Patients were selected upon satisfying three conditions: (1) they had a pre-operative knee computed tomography (CT) scan; (2) they would have the Oxford UKA (Biomet Ltd, Swindon, UK) on their medial condyle; (3) they lived within 10 miles of the hospital. The patients were recruited regardless of whether cemented or cementless fixation would be used.

The first DXA scan was performed within 10 days from the date of surgery, with the remaining scans carried out at 3, 6, and 12 months. Patients were scanned in both AP and lateral knee orientations.

All DXA scans were performed using a GE Lunar Prodigy Scanner (GE Healthcare, Chalfont St Giles, UK). AP and lateral scans were performed using wooden limb-positioning jigs specially made for the study. The AP scan was taken with the tibia inclined at 7° to the scanner bed so that a vertical X-ray beam would be approximately parallel to the tibial plateau, while the lateral scan was taken at 30° knee flexion. Since the scanner did not have a pre-defined setting for knee scans, the ‘AP Spine’ mode was selected with ‘Smart Scan’ mode setting deactivated. As is commonly used for knee scans [25], two rice bags were also used as a soft tissue substitute.

The reproducibility of the BMD measurements was calculated in each subject by carrying out two consecutive scans at 6 months in both AP and lateral projections, with the subject being repositioned between scans. The test–retest error was calculated as follows:

$${\text{Error}}\,(\%) = \frac{1}{N}\mathop \sum \limits_{n = 1}^{N} \frac{{\sqrt {\left( {{\text{BMDi}}_{n} - {\text{BMDii}}_{n} } \right)^{2} } }}{{\frac{1}{2}\left( {{\text{BMDi}}_{n} + {\text{BMDii}}_{n} } \right)}}$$

where BMDi is the first BMD reading of patient n, BMDii is the second reading of patient *n*, and *N* is the total number of patients. A standard Lunar calibration block was used for daily quality assurance of the scanner, and at regular intervals, a secondary calibration check was completed using an aluminium spine phantom.

**Fig. 1:**
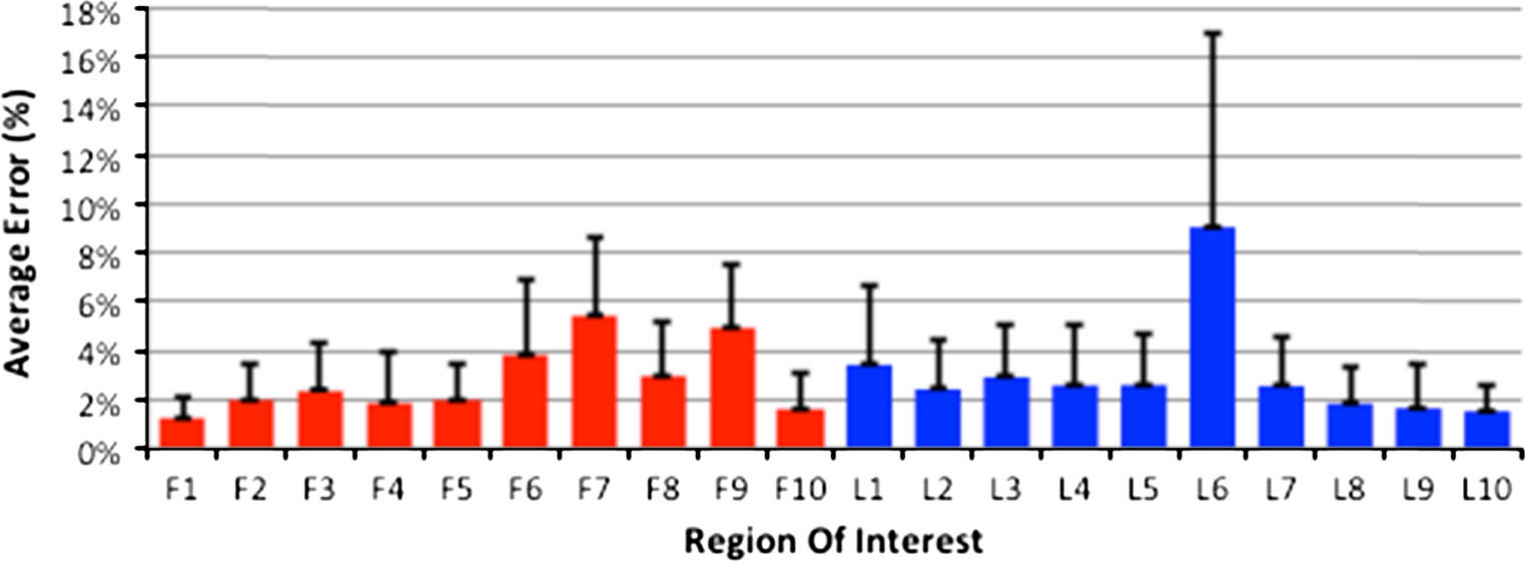
Average test–retest error of the DXA BMD measurements for each ROI. ROIs F1–10 are AP scan and L1–10 are lateral scan ROIs (mean + SD, *n* = 11)

Figure 1 shows the errors associated with patient repositioning. The errors for ROI F7 and F8 were larger because they were sensitive to the medial position of the patella: BMD was higher when the patella was medial and overlapping ROI F7 and F8. The high error of ROI L6 occurred because the BMD was sensitive to the position of the fibula. In addition, baseline data for one knee were unavailable in the lateral view.

The patient data were anonymised and analysed using EnCore 2008 (GE Healthcare, Chalfont St Giles, UK). The scans were converted to ‘knee’ mode, and ten regions-of-interest (ROI) were defined for the AP (ROI F1–10) and lateral (ROI L1–10) scans. All the 1-year data were analysed at the same time by a single user to ensure consistency.

### Statistical analysis

The Kolmogorov–Smirnov test was used to test all variables for normality using SPSS software (IBM Software Group, New York, USA). The test confirmed that all BMD variables were normally distributed and that a paired Student *t* test was suitable for testing statistical difference. A power analysis was not done in view of the ethics permit only being for the small number of cases.

## Results

Thirteen patients consented to join this study, but one patient dropped out immediately after the first scan, due to discomfort in other joints during the scanning procedure, and another was lost prior to the scan at 12 months, leaving 11 patients. The data relate to seven UKA patients with cement fixation (two male and five female, aged 59 ± 12 years (mean ± SD), range 42–79 years) and four patients with cementless fixation (four male, aged 69 ± 8 years, range 61–79 years).

### Tibia

**Fig. 2:**
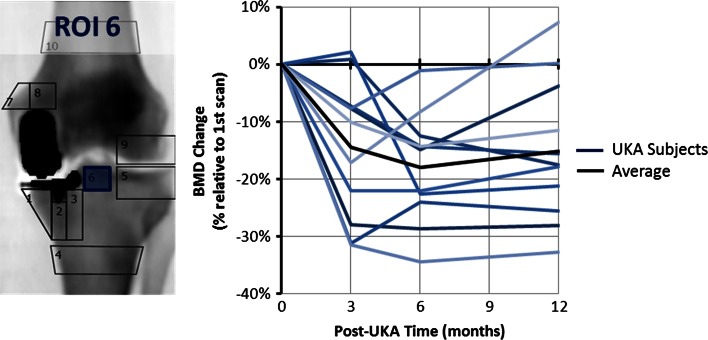
BMD changes post-UKA at ROI F6. A significant drop of BMD was observed at 6 months and 1 year

Figure 2 presents the BMD changes beneath the tibial intercondylar eminence (ROI F6) up to 1 year post-arthroplasty; the BMD drop was significant at 6 months (*P* = 0.0001) and at 1 year (*P* = 0.0022).

**Fig. 3:**
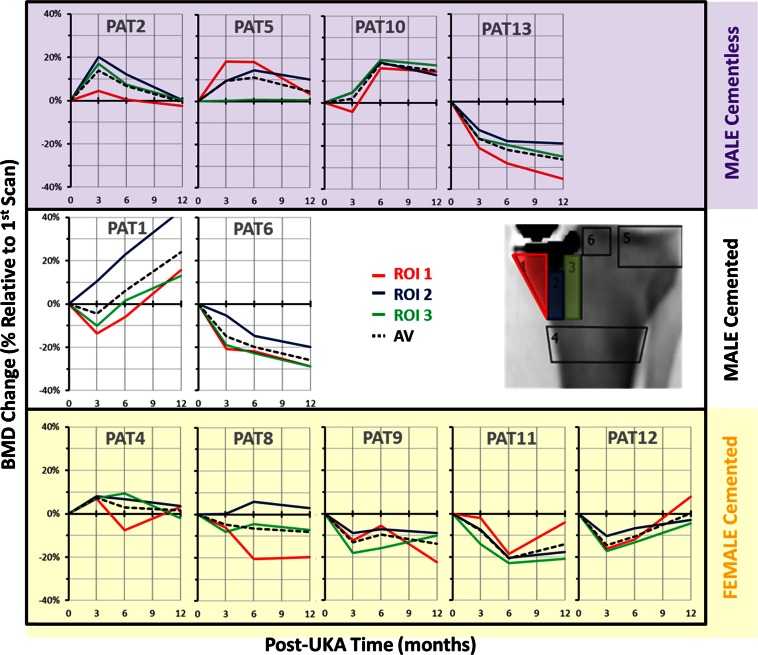
Anteroposterior scan BMD changes under the tibial tray of each patient

**Fig. 4:**
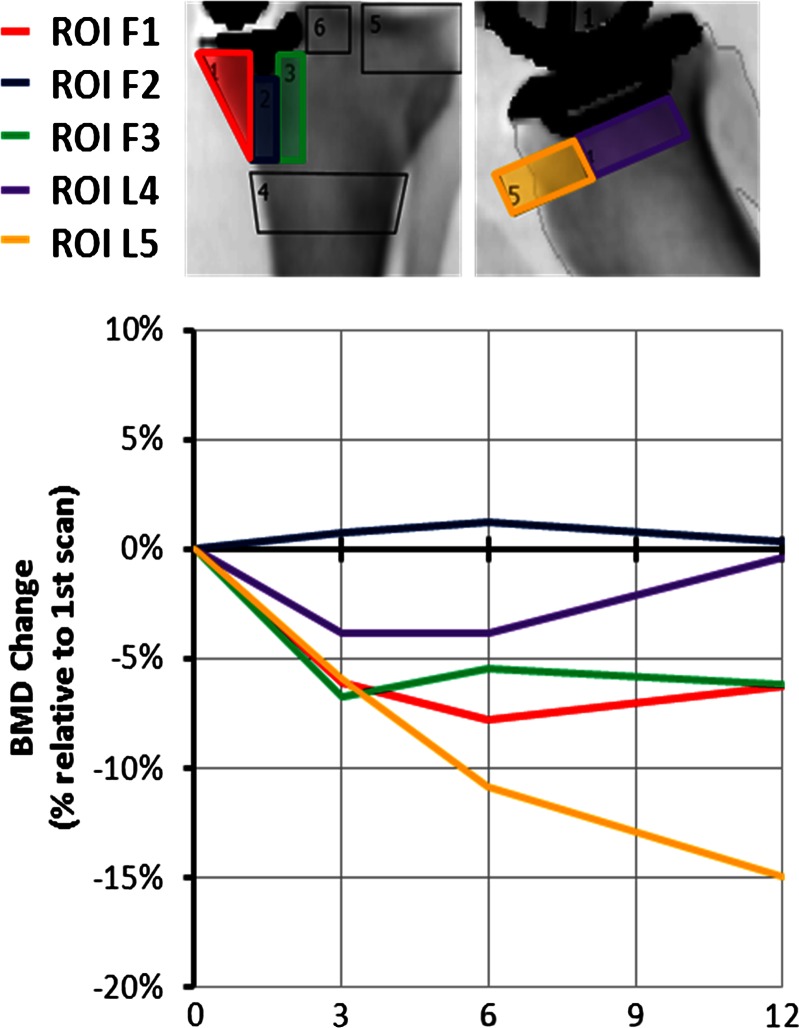
Mean BMD changes under UKA tibial implants; *n* = 11

Figure 3 displays the BMD changes at three ROI located beneath the UKA tibial tray: there was a considerable variation between the subjects. The total average change in BMD under the tibial tray at 1 year was −4 ± 17 %. Figure 4 shows the average BMD changes in the proximal tibia, with no mean change under the keel (ROI F2) and small mean losses (6 %) in the regions medial (ROI F1) and lateral to the keel (ROI F3).

**Fig. 5:**
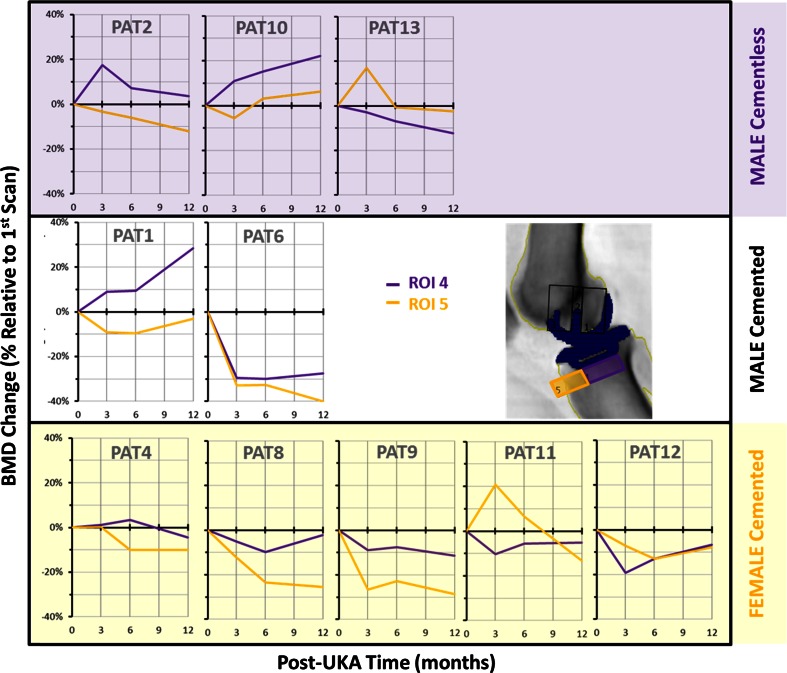
Lateral scan BMD changes under the tibial tray for each patient

From the lateral view (Fig. 5), the mean BMD under the keel was stable, while it decreased significantly (*P* = 0.0126) in the anterior region.

### Femur

**Fig. 6:**
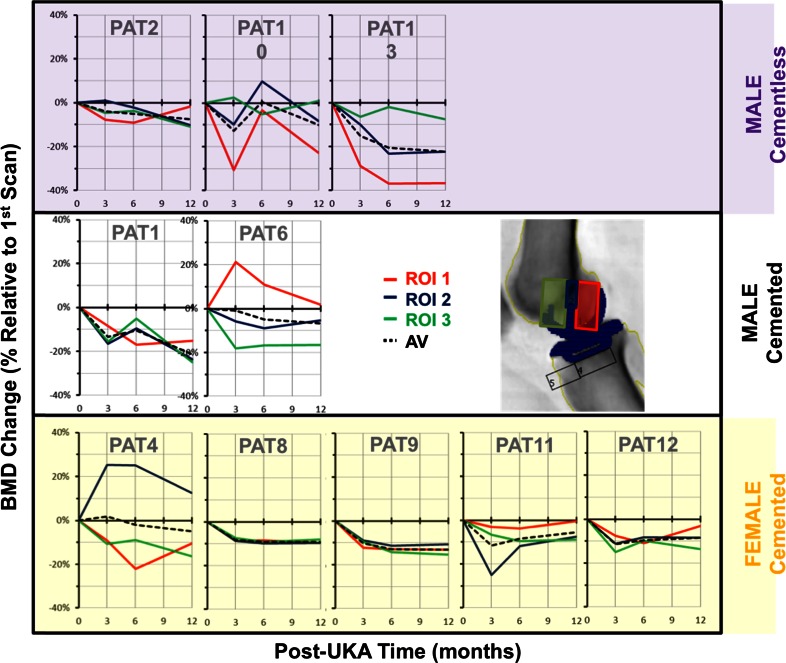
Lateral scan BMD changes under the femoral component for each patient

**Fig. 7:**
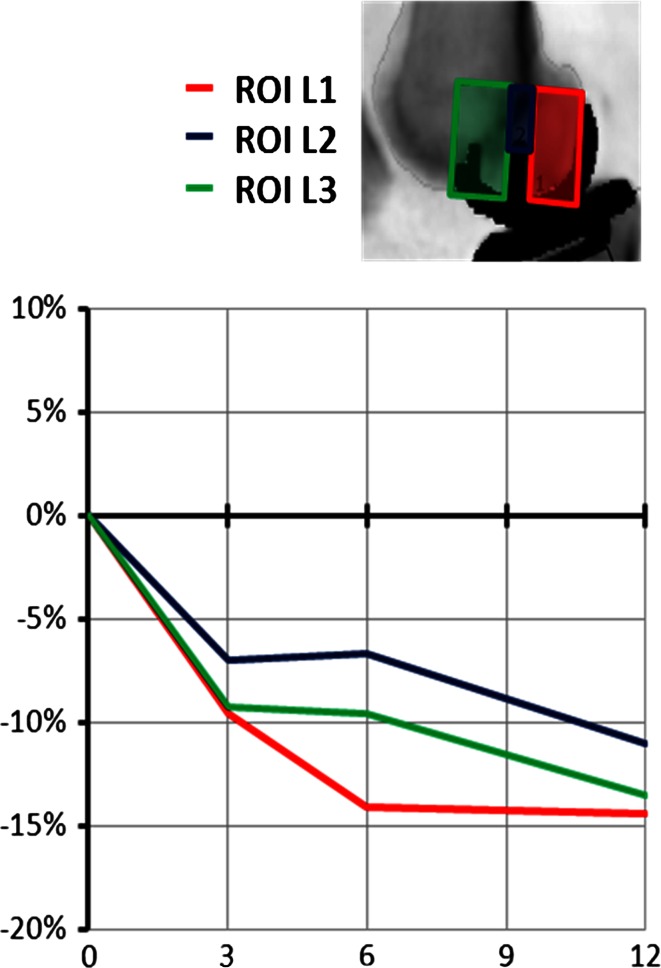
Mean BMD changes under UKA femoral implants; *n* = 11

Figure 6 displays the BMD changes at three ROI located beneath the UKA femoral component of all subjects in the study, again showing considerable variability of behaviour. The average BMD decreased significantly under the central peg (*P* = 0.0104), under the posterior of the implant (*P* = 0.0004), and anteriorly (*P* = 0.0004) (Fig. 7).

### Effect of implant fixation method

**Fig. 8:**
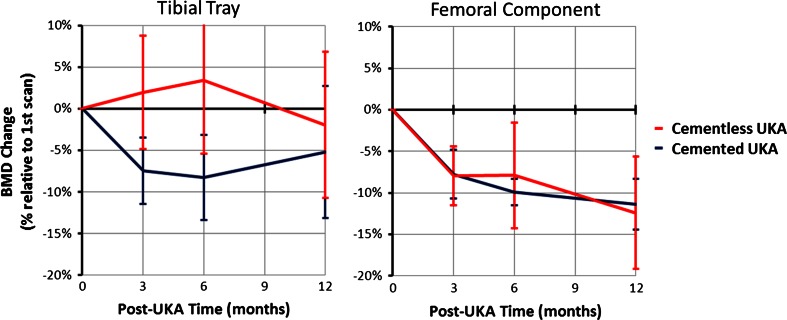
BMD changes for cemented and cementless UKAs, tibial tray (*left*) and femoral component (*right*) (mean + SD)

The mean bone changes in cemented and cementless fixation beneath both the tibial tray and femoral components were similar, but with considerable variation between patients (Fig. 8).

### Overall

Despite variability between individual patients, the mean reductions in BMD in the femur (ROI L1–L3; Fig. 8) showed a more consistent pattern overall than did those in the tibia (Fig. 4 ).

## Discussion

The most important findings of this study were the extent of the loss of BMD, as hypothesised, but also the variability of the BMD changes, post-UKA, in both the tibia and femur, with standard deviations reaching 30 %. The biggest mean reduction occurred under the tibial intercondylar eminence, perhaps explaining why early subsidence of the tibial tray can occur at its lateral aspect, tipping into valgus [11]. The mean bone loss under the tibial tray was 2 %, which is equivalent to the overall bone loss that occurred in the whole knee (2–5 %). However, the mean bone loss at the anterior portion was higher, reflecting that the single-radius femoral component reduces anterior loading in extension. Comparison with a fixed-bearing polyradial femoral design would be interesting. The mean bone loss under the femoral component averaged 13 %.

Most subjects saw a large decline in BMD in the first 6 months following surgery, followed by a recovery. This trend is common following TKA [9]. The large loss of BMD below the tibial eminence may have occurred due to inactivity or deficiency of the ACL [13]. Another DXA study of UKA [24] found similar loss of femoral bone density within the first year and then no significant change up to 7 years post-surgery. Unlike the present study, they reported that the medial tibia gained bone density post-UKA, and did not find much variability of the BMD changes among individuals.

An overall decline in BMD occurs in normal subjects with age [6] and has been similarly reported post-TKA [9, 22] and UKA [11]. In this study, the BMD in the tibial and femoral diaphyses declined by a mean of 2 ± 4 % during the year.

Similar BMD changes to those in this study have also been seen below TKA tibial trays, with bone loss in the first 3 months for cemented fixation [10, 13] and bone gain for cementless fixation [3]. The decision to use cemented or cementless implants was not randomised in this study: surgeons tend to use cementless implants on denser (‘stronger’) bone, which is a judgement made based on experience. This suggests that the cementless group would naturally respond better to UKA, and that has been supported by evidence from TKAs [8].

There were large differences in bone response between subjects, which is a common characteristic of post-arthroplasty DXA studies [3, 10, 13, 22]. Hormonal status and nutritional status are known to impact on bone healing, and genetic responsiveness to the stress of surgery may contribute to this variation.

The results of this study must be considered with regard to its limitations. The sample size of 11 subjects was small, and although some conclusions regarding the bone response after UKA are possible, conclusions comparing cemented versus cementless fixation are not appropriate because of the small subgroups, which were not randomised. Larger numbers of each fixation type are required before more definitive statements or correlation with activity levels can be made. The raw data allowed the qualitative observation that the males with cementless implants tended to have higher bone density at the start and end of the study, while some cemented cases had lower bone density at both the start and end of the study. The accuracy and precision of DXA for the evaluation of bone density in the proximity of metal TKA implants was assessed by Robertson et al. [21], who showed that DXA was better than the other methods considered. DXA has a reported precision of 0.9–8.3 % when applied to TKA [10, 15, 25], similar to the present study.

Overall BMD reductions under the prosthetic components were low with either cemented or cementless fixation. However, the variability of responses between patients means that the bone changes may be a concern for some patients. With variations in the size reported, poor outcome of a device in a specific patient may have more to do with the individual’s response to surgery than to the design of the prosthesis.

## Conclusion

This study found significant loss of BMD under the components of UKA during the year post-implantation. There was also a large amount of individual variation in the bone responses. This study had insufficient numbers to identify differences between cemented and cementless designs.
